# Nontargeted metabolomics to characterize the effects of isotretinoin on skin metabolism in rabbit with acne

**DOI:** 10.3389/fphar.2022.963472

**Published:** 2022-08-31

**Authors:** Xiao-Liang Ou-Yang, Deng Zhang, Xiu-Ping Wang, Si-Min Yu, Zhen Xiao, Wei Li, Chun-Ming Li

**Affiliations:** ^1^ Department of Dermatology, The Second Affiliated Hospital of Nanchang University, Nanchang University, Nanchang, China; ^2^ Department of Dermatology, Taiyuan Central Hospital of Shanxi Medical University, Shanxi Medical University, Taiyuan, China; ^3^ Department of Plastic Surgery, The Second Affiliated Hospital of Nanchang University, Nanchang University, Nanchang, China

**Keywords:** acne vulgaris, metabolomics, isotretinoin, UHPLC-QTOF-MS, animal model

## Abstract

**Background:** Acne vulgaris is a chronic inflammatory disease of the pilosebaceous unit. This study aimed to explore the pathogenesis of acne and the therapeutic mechanism of isotretinoin from the metabolic perspective in coal tar-induced acne in rabbits.

**Methods:** Ultra-high performance liquid chromatography/quadrupole time-of-flight mass spectrometry (UHPLC-qTOF-MS) based metabolomics was used to identify skin metabolites in groups C (blank control), M (model group) and T (isotretinoin group). Multivariate statistical analysis was used to process the metabolomics data.

**Results:** 98 differential metabolites in group C and group M were identified. The highest proportion of differential metabolites were organic acids and derivatives, lipid metabolites, organic heterocyclic compounds, and nucleoside metabolites. The most significant metabolic pathways included protein digestion and absorption, central carbon metabolism in cancer, ABC transporters, aminoacyl-tRNA biosynthesis, biosynthesis of amino acids, and sphingolipid signaling pathway. Isotretinoin treatment normalized eight of these metabolites.

**Conclusions:** Our study will help to further elucidate the pathogenesis of acne, the mechanism of isotretinoin at the metabolite level, and identify new therapeutic targets for treating acne.

## 1 Introduction

Acne vulgaris is a chronic inflammatory disease of the pilosebaceous unit and occurs most prominently at skin sites with a high density of sebaceous glands, such as the face, chest, and back (Poli et al., 2020). As this disease affects 85% of adolescents (Habeshian and Cohen, 2020), it is the most common dermatosis. Anxiety, depression, somatization, and in rare cases, psychosis have been reported in patients with acne (Stamu-O'Brien et al., 2021). Acne pathogenesis has implicated four major factors: increased sebum production, altered keratinization processes leading to comedone formation, follicular colonization by Propionibacterium acnes (P.acnes), and inflammatory mediators around the pilosebaceous unit (Hazarika, 2021). Recent advances have further elucidated that metabolic factors also play an essential role in the development of acne (Zouboulis, 2020).

Metabolites are small-molecule chemicals produced by endogenous catabolism or anabolism. Metabolomics uses advanced analytical chemistry techniques to characterize metabolites from cells, organs, tissues, or biological fluids in high throughput and is a newly developed technique after genomics, transcriptomics, and proteomics (Wishart, 2019). It is mainly used for disease etiology and pathogenesis, clinical diagnosis, clinical drug use guidance, and preclinical animal model screening. At present, metabolomics has been applied in many dermatologic diseases, such as psoriasis (Pohla et al., 2020), atopic dermatitis (Hashimoto et al., 2019), melanoma (Abaffy et al., 2013), and basal cell carcinoma (Mun et al., 2016).

Isotretinoin is FDA approved for treating severe recalcitrant acne vulgaris and is also recommended for moderate acne that is treatment-resistant, leads to scarring, or causes significant psychosocial distress (Habeshian and Cohen, 2020). It acts by conversion to all-trans retinoic acid, which penetrates the cell nucleus and binds to nuclear receptors of two families: Retinoic Acids Receptors and Retinoids X Receptors (Bagatin and Costa, 2020). Isotretinoin is thought to improve acne by reducing the size of sebaceous glands and sebum production, normalizing follicle keratinization, inhibiting the growth of P.acnes by altering the follicular environment, and reducing inflammation. Hence, it is the only drug that targets all the major factors associated with acne (Fallah and Rademaker, 2021).

In this study, we analyzed the skin metabolic profile of coal tar-induced acne in rabbits using a nontargeted metabolomics approach based on ultra-high performance liquid chromatography/quadrupole-time-of-fight mass spectrometry (UHPLC-qTOF-MS) and compared the changes of metabolic profile before and after isotretinoin treatment. The study aimed to determine the skin metabolic profile of coal tar-induced acne in rabbits and investigate the mechanism of isotretinoin in treating acne at the metabolomic level.

## 2 Materials and methods

### 2.1 Chemicals and reagents

P.acnes ATCC 6919 was obtained from the Guangdong Microbial Culture Collection Center. Columbia Blood Agar Base and Brain Heart Infusion Broth were purchased from Hopebio. Ammonium acetate (NH_4_AC), ammonium hydroxide (NH_4_OH), ammonium fluoride (NH_4_F), and formic acid (FA) were purchased from Sigma Aldrich. Acetonitrile was purchased from Merck.

### 2.2 Experimental animals

Thirty 3-month-old New Zealand white rabbits (1.7–2.5 kg, Ganzhou, China) were used for animal experiments. All animals were fed with standard feed in a single cage to avoid scratching and damaging their ears. The room environment was controlled at a temperature of 20–26°C, the humidity of 40%–70%, and day and night time of 12 h each, with free access to water and food. The animals were acclimatized to their surroundings for at least 1 week before the experiment to reduce stress. All animal procedures were performed following the Guidelines for Care and Use of Laboratory Animals issued by the China Association of Laboratory Animal Care and were approved by the Research Ethical Committee of The Second Affiliated Hospital of Nanchang University (Nanchang, China).

### 2.3 P. acnes

P.acnes (ATCC 6919) were grown on Columbia Blood Agar Base and incubated under anaerobic conditions using Brain Heart Infusion Broth at 37°C for 72 h. Bacteria were suspended in the appropriate amount of PBS for use in experiments, and the concentration of bacteria was adjusted to 6 ×10^8^ CFU/ml.

### 2.4 Coal tar-induced acne in rabbits

The animals were randomly divided into three groups: group C (blank control group, *n* = 10), group M (model group, *n* = 10), and group T (isotretinoin group, *n* = 10). Group C served as a blank control. 2% coal tar (CAS: 8007-45-2, Alfa Aesar), 0.5 ml/d, was applied uniformly to the central inner ear of each rabbit in groups M and T, to the extent of approximately 2 cm × 2 cm, for a total of 14 days. 100 μl of bacteria solution was injected intradermally at multiple points (6 points) in the inner ear starting on day 7, every other day, for a total of 4 times. 20 mg/kg/d of oral isotretinoin was given to group T from day 1.

### 2.5 Skin samples

Rabbits were executed by air embolization on day 14, ear thickness was measured before sampling, and 2 cm × 2 cm skin tissue was cut from the lesion and divided into two copies of 1 cm × 2 cm tissue. One of the skin samples was fixed with 4% paraformaldehyde, paraffin-embedded, and tissue sections (5 μm) were prepared according to standard procedures, stained with hematoxylin-eosin (H&E), and light microscopy was performed for pathological observation. Another skin sample was snap frozen in liquid nitrogen and stored at −80°C until being used for metabolic analysis.

### 2.6 Skin sample preparation for metabolic analysis

10 mg skin samples were cut on dry ice into an Eppendorf tube. The tissue samples with 200 μL of H_2_O were homogenized using the homogenizer. 800 μL methanol/acetonitrile was added to the homogenized solution for metabolite extraction. The mixture was centrifuged for 15 min to extract the supernatant. The supernatant was dried in a vacuum centrifuge. The samples were re-dissolved in 100 μl acetonitrile/water solvent when LC-MS analysis.

Quality control (QC) samples were prepared by pooling 10 μl of each sample and analyzed with the other samples. The QC samples were inserted in every 5 samples to monitor the stability and repeatability of instrument analysis.

### 2.7 LC-MS/MS analysis

Analyses were performed using a UHPLC (1290 Infinity LC, Agilent Technologies, Santa Clara, CA, United States) coupled to a quadrupole time-of-flight (AB Sciex TripleTOF 6600, Framingham, MA, United States).

For HILIC separation, samples were analyzed using a 2.1 mm × 100 mm ACQUIY UPLC BEH 1.7 µm column (waters, Ireland). In both Electron Spray Ionization (ESI) positive and negative modes, the mobile phase contained A = 25 mM ammonium acetate and 25 mM ammonium hydroxide in water and B = acetonitrile. The gradient was 85% B for 1 min, linearly reduced to 65% in 11 min, then reduced to 40% in 0.1 min and kept for 4 min, then increased to 85% in 0.1 min, with a 5 min re-equilibration period employed.

The ESI source conditions were set as follows: Ion Source Gas1 (Gas1) as 60, Ion Source Gas2 (Gas2) as 60, curtain gas (CUR) as 30, source temperature: 600°C, IonSpray Voltage Floating (ISVF) ± 5500 V. In MS only acquisition, the instrument was set to acquire over the m/z range 60–1000 Da, and the accumulation time for TOF MS scan was set at 0.20 s/spectra. In auto MS/MS acquisition, the instrument was set to acquire over the m/z range 25–1000 Da, and the accumulation time for product ion scan was set at 0.05 s/spectra. The collision energy (CE) was fixed at 35 V with ± 15 eV; declustering potential (DP), 60 V (+) and −60 V (−); exclude isotopes within 4 Da, candidate ions to monitor per cycle: 10.

### 2.8 Data processing

The raw MS data (wiff.scan files) were converted to MzXML files using ProteoWizard MSConvert before importing them into freely available XCMS software. For peak picking, the following parameters were used: centWave m/z = 25 ppm, peak width = c (10, 60), prefilter = c (10, 100). For peak grouping, bw = 5, mzwid = 0.025, minfrac = 0.5 were used. CAMERA (Collection of Algorithms of MEtabolite pRofile Annotation) was used for annotation of isotopes and adducts. Only the variables with more than 50% of the nonzero measurement values in the extracted ion features were kept in at least one group.

Compound identification of metabolites was performed by comparing accuracy m/z value (<10 ppm) and MS/MS spectra with an in-house database established with available authentic standards. The structural identification of metabolites in biological samples was performed by comparing with the information of retention time, molecular mass (molecular mass error <10ppm), secondary fragmentation spectra and collision energy of metabolites in the database, and the identification results were strictly checked and confirmed by manual secondary inspection. The identification level is above level 2. Level 2 is defined as matched to literature data or databases by diagnostic evidence, at least two orthogonal pieces of information, including evidence that excludes all other candidates.Receiver operating characteristic (ROC) curves (Graphpad 8.0) were then applied to analyze the data to assess the predictive power of the identified biomarkers.

Metabolites were compared with free online databases KEGG (http://www.genome.jp/kegg/) and HMDB (http://www.hmdb.ca/), and the corresponding KEGG pathways were extracted. KEGG enrichment analysis was performed using MetaboAnalyst (www.metaboanalyst.ca).

### 2.9 Statistical analysis

After normalized to total peak intensity, the processed data were analyzed by R package (ropls), where it was subjected to multivariate data analysis, including orthogonal partial least-squares discriminant analysis (OPLS-DA) and Partial Least Squares Discriminant Analysis (PLS-DA). The variable importance (VIP) in the projection value of each variable in the OPLS-DA model was calculated to indicate its contribution to the classification. Metabolites with the VIP value > 1 were further applied to Student’s t-test at the univariate level to measure the significance of each metabolite, the *p* values less than 0.05 were considered statistically significant.

## 3 Results

### 3.1 Histological changes in the skin of the rabbit models

**FIGURE 1:**
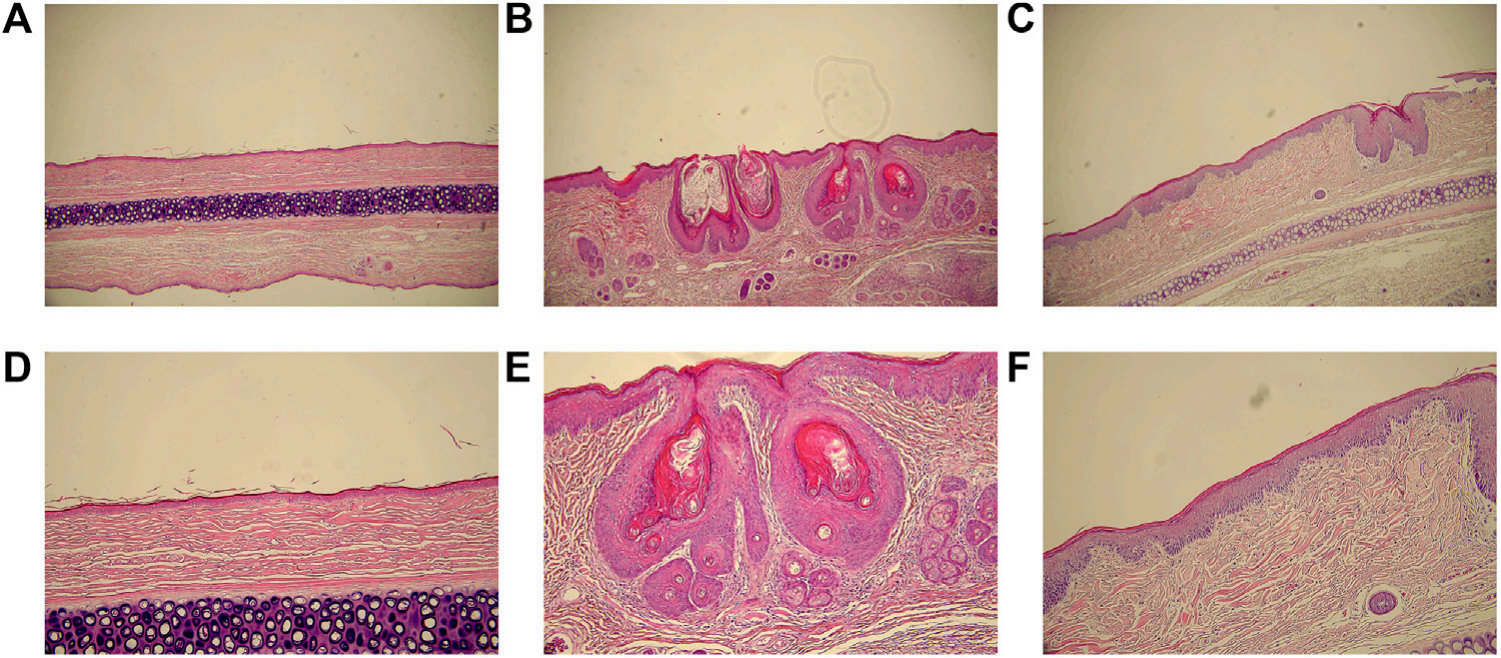
The histological analyses of skin lesions (H&E staining, ×200 and ×100). **(A)**, **(B)** and **(C)**: magnification ×100. **(D) (E)** and **(F)**: magnification ×200. **(A)** and **(D)**: group C; **(B)** and **(E)**: group M; **(C)** and **(F)**: group T.

In group M, acne-like and papular lesions appeared on the skin of rabbit ears. Skin histological analysis of group M (Figures 1B,E) showed hyperplasia of the compound squamous epithelium with marked thickening of the stratum corneum, dermal congestion, inflammatory cell infiltration, and significantly increased sebaceous glands size. Hyperkeratosis of follicular sebaceous glands and obstruction of follicular pores were observed as fundamental pathological mechanisms in the development of acne. In group T (Figures 1C,F), keratosis was improved, inflammatory cell infiltration was reduced, and sebaceous glands became smaller, which suggested that isotretinoin significantly reduced the inflammation of the skin.

### 3.2 Skin metabolic profile changes in coal tar-induced acne in rabbits

**FIGURE 2:**
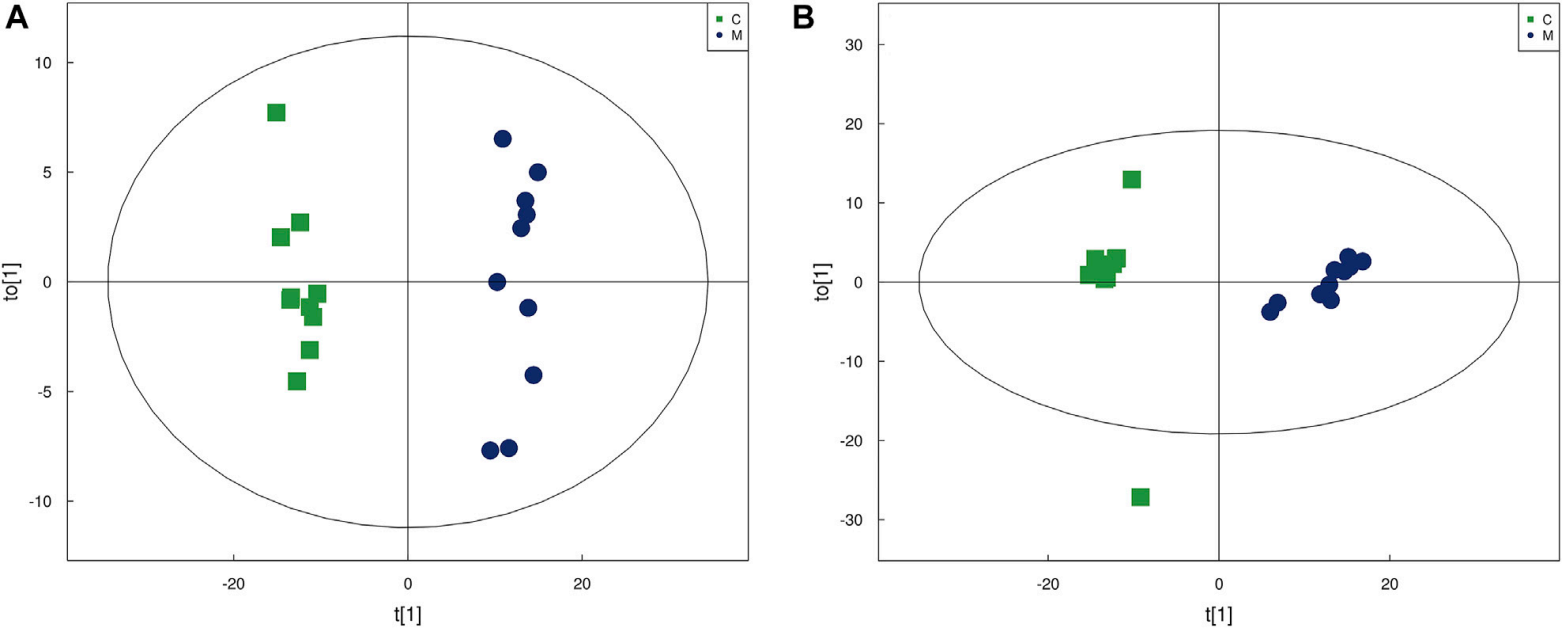
The score plots from OPLS-DA mode. **(A)** OPLS-DA score plots in positive modes of the group C and group M. **(B)** OPLS-DA score plots in negative mode of the group C and group M.

OPLS-DA is a supervised discriminant analysis statistical method. At the same time, VIP was calculated to measure the impact strength and interpretation ability of each metabolite expression pattern on the classification and discrimination of each group of samples, thus assisting the screening of marker metabolites. In the OPLS-DA score chart (Figure 2), the skin samples of group C and group M were closely clustered in both positive and negative modes. Besides, an obvious distinction between the two groups could be observed, which indicated significant changes in the skin metabolic profile of the acne model.

### 3.3 Identification of potential biomarkers and metabolic pathways

**FIGURE 3:**
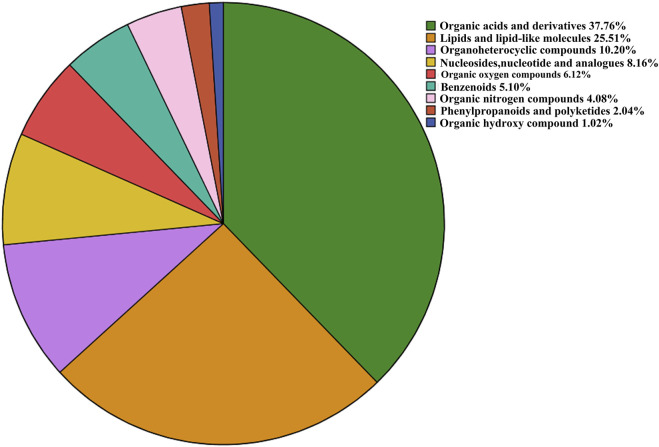
Proportion of identified metabolites in each chemical classification.

**FIGURE 4:**
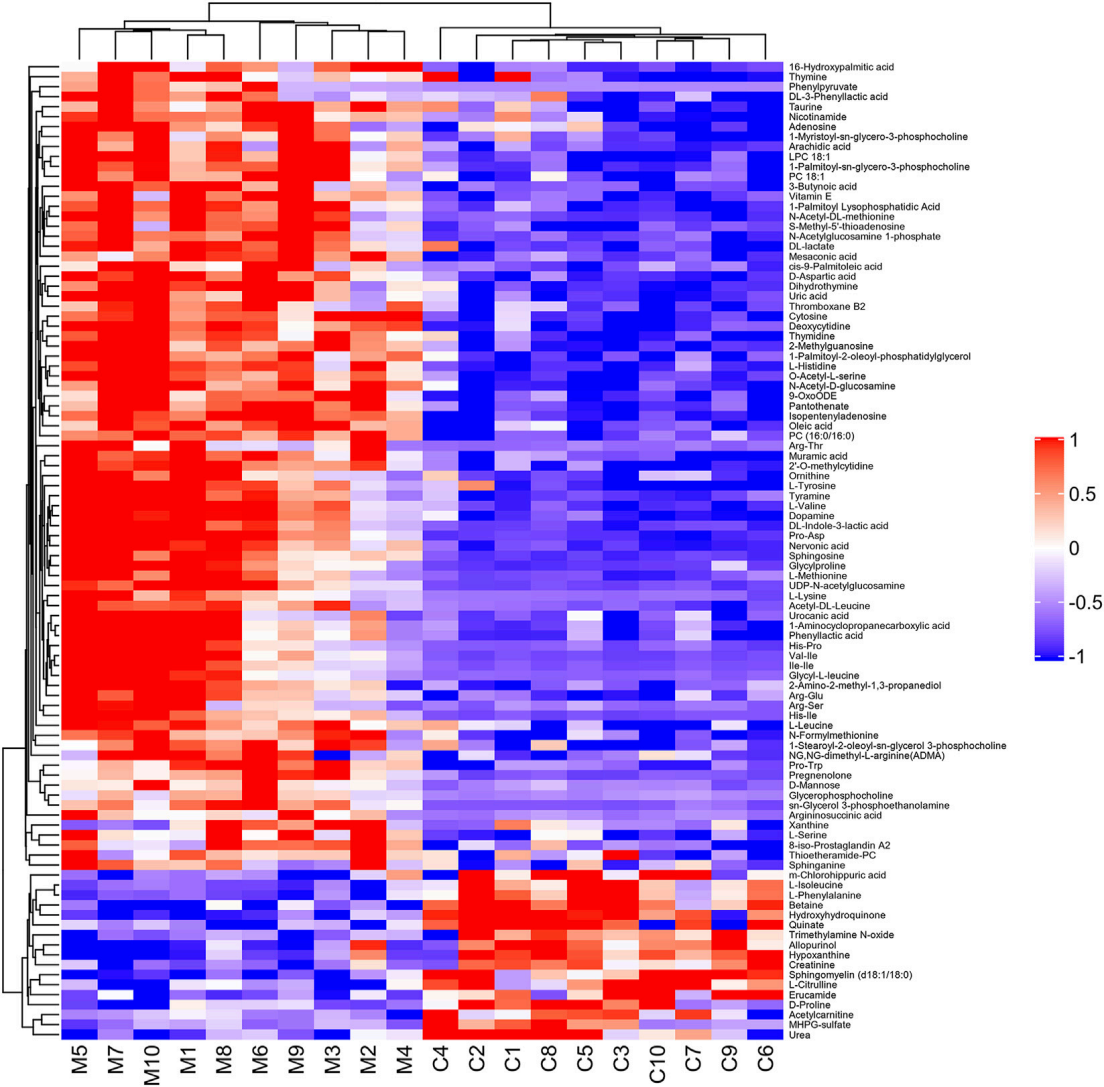
Hierarchical clustering heat map of 98 differential metabolites between group C and group M. The values of differential metabolites were normalized and shown as a color scale. Red represents significant upregulation, blue represents significant downregulation, and the color scale indicates the level of metabolites.

**FIGURE 5:**
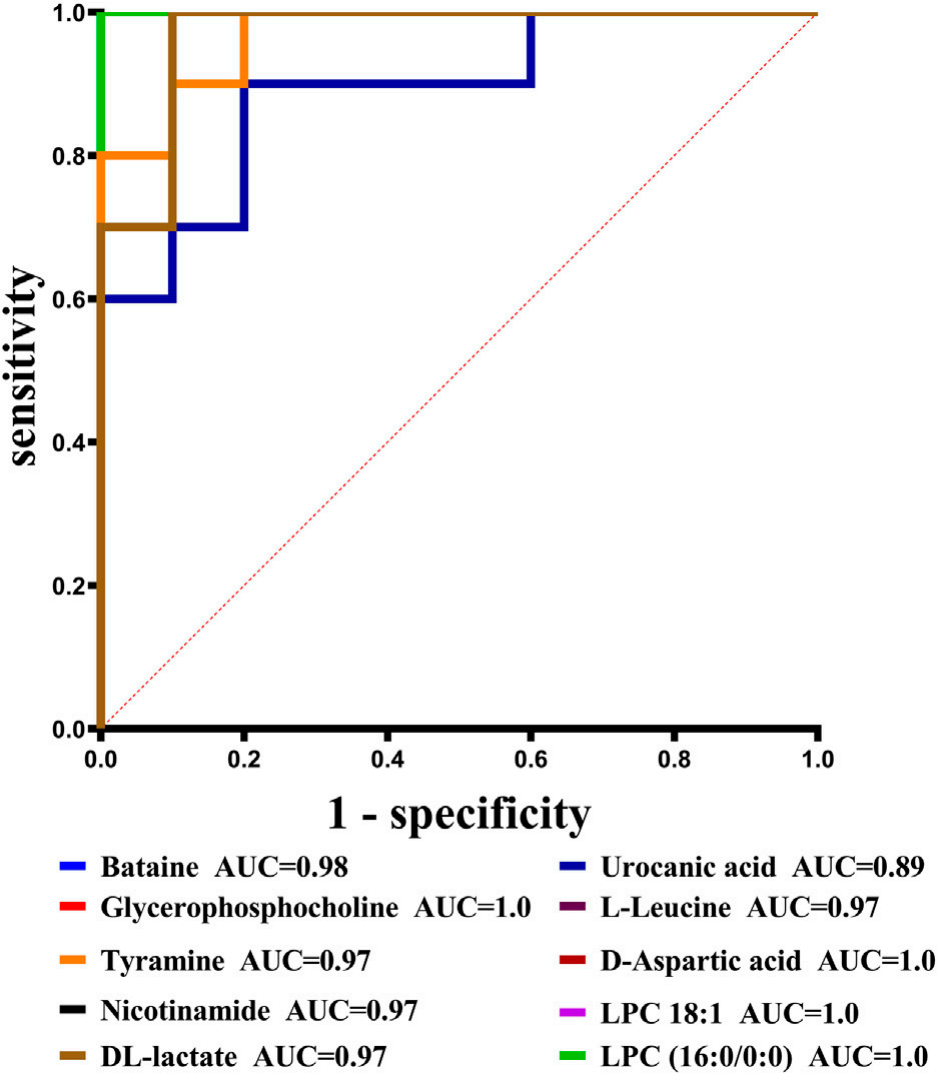
ROC curves for potential biomarkers with VIP values in the top 10.

With VIP > 1.0 in the OPLS-DA models, fold change (FC)≥1.2 or FC ≤ 0.8, and *p* < 0.05, 98 differential metabolites (70 in positive and 28 in negative modes, the detailed information was shown in the Supplementary Table S1) in group C and group M were identified. The chemical classification attribution map (Figure 3) revealed that the highest proportion of differential metabolites were organic acids and derivatives (37 in total, including 34 amino acid metabolites), lipid metabolites, organic heterocyclic compounds, and nucleoside metabolites. In group M, 17 metabolites decreased, while the other 81 metabolites increased. Hierarchical clustering heat map (Figure 4) showed that skin metabolites in group C were significantly separated from those in group M, indicating a considerably altered skin metabolic profile in rabbits with acne. The ROC curves were used to assess the predictive value of the screened potential markers. The ROC curve plots for metabolites with VIP values in the top 10 were shown in Figure 5, and ROC curves analysis for each biomarker were shown in Supplementary Table S2. AUC values between 0.7 and 0.9 have “medium” accuracy, and values greater than 0.9 have “high” accuracy. The results indicated that the identified markers have strong diagnostic performance.

**FIGURE 6:**
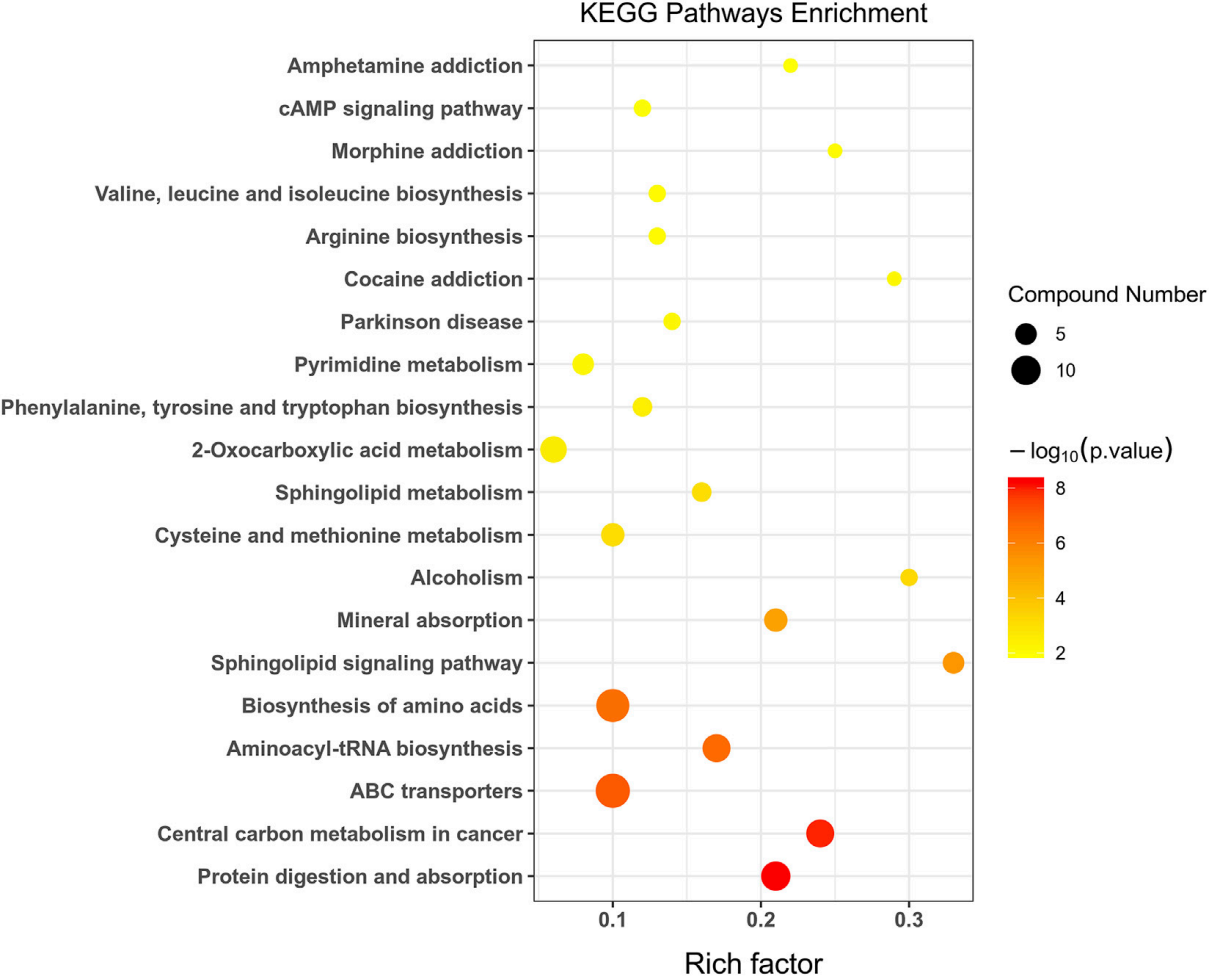
Statistics of KEGG enrichment. The x axis indicates the rich factor corresponding to each pathway, and the y axis indicates name of the KEGG metabolic pathway. The size and color of bubbles represent the number and degree of enrichment of different metabolites, respectively.

Fisher’s exact test was used to analyze and calculate the significance level of metabolite enrichment for each pathway to identify significantly affected metabolic and signal transduction pathways. The smaller the *p*-value, the more significant the difference in the metabolic pathway. A total of 32 metabolic pathways were significantly changed (*p* < 0.05) in group C and group M (Figure 6). These included six amino acid metabolic pathways, two lipid metabolic pathways, six nucleotide metabolic pathways, and two signal transduction pathways. Basic metabolic pathways such as protein digestion and absorption, central carbon metabolism in cancer, ATP-binding cassette (ABC) transporters, and amino-acyl-tRNA biosynthesis were also involved. The most significant metabolic pathways included protein digestion and absorption, central carbon metabolism in cancer, ABC transporters, aminoacyl-tRNA biosynthesis, biosynthesis of amino acids, and sphingolipid signaling pathway, as shown in Supplementary Table S3.

### 3.4 Metabolites changes after isotretinoin treatment

**FIGURE 7:**
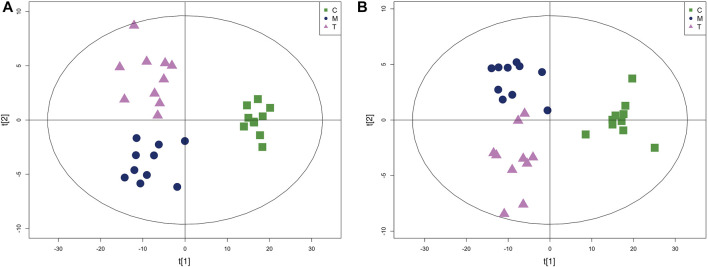
The PLS-DA score plot of group C, group M and group T in skin tissue metabolism profile. **(A)** Positive mode and **(B)** Negative mode.

**FIGURE 8:**
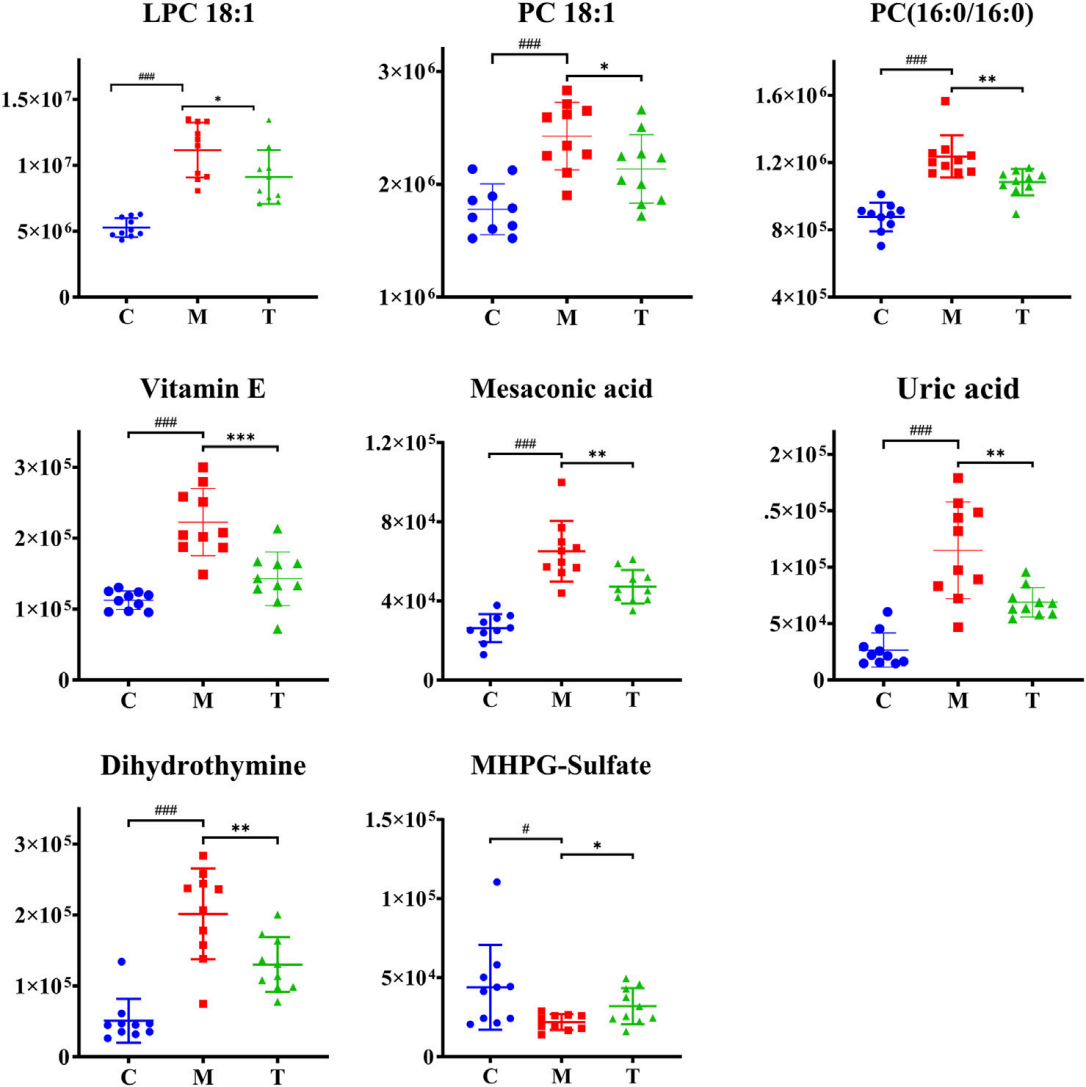
Scatter graph of metabolites changes in coal tar-induced acne in rabbits treated by isotretinoin. Group C vs. group M, #*p* < 0.05, ##*p* < 0.01, ###*p* < 0.001; group M vs. group T, *p < 0.05, **p < 0.01, ***p < 0.001.

PLS-DA analysis can enhance the identification of variables contributing more to the classification and obtain a more desirable separation between groups. PLS-DA score plots for the three groups of skin samples were shown in Figure 7. A clear separation of the metabolic profiles of the three groups was observed, suggesting that oral administration of isotretinoin led to a significantly changed metabolic profile. We found that isotretinoin treatment normalized eight metabolites in the acne model (Figure 8). These eight metabolites included LPC 18:1, PC 18:1, PC (16:0/16:0), Vitamin E, Mesaconic acid, Uric acid, Dihydrothymine, and MHPG-sulfate. Five of these metabolites, namely LPC 18:1, PC 18:1, PC (16:0/16:0), alpha-Tocopherol, and Mesaconic acid, are Lipids and lipid-like molecules.

## 4 Discussion

In this study, we investigated the mechanism of isotretinoin in treating coal tar-induced acne model by metabolomics. We demonstrated that 98 metabolites were markedly altered in group M compared to group C. They consisted mainly of amino acid and lipid metabolites. Meanwhile, a total of 32 metabolism pathways were perturbed. Furthermore, we identified that isotretinoin normalized eight of these metabolites.

### 4.1 Significant metabolic pathways in the development of acne

#### 4.1.1 Protein digestion and absorption and biosynthesis of amino acids

Amino acid plays a vital role in immune and inflammatory reactions, synthesizing proteins involved in immune cell proliferation and immune responses (Prochnicki and Latz, 2017). In our study, protein digestion and absorption and biosynthesis of amino acids metabolic pathways were enriched. Notably, these pathways were associated with amino acid metabolism. Nine proteinogenic amino acids were involved in these two pathways, seven amino acids were elevated (L-Lysine, L-Serine, L-Methionine, L-Tyrosine, L-Histidine, L-Valine, L-Leucine), and two amino acids were decreased (L-Phenylalanine, L-Isoleucine). Histidine and serine play an essential role in preserving the moisture of the stratum corneum of the epidermis, while Lysine is critical for proper collagen and elastin functions in the dermis. Besides, isoleucine, leucine, and valine are branched-chain amino acid. Amino acid mixtures, including Isoleucine, leucine, and Valine, significantly increased dermal collagen synthesis in hairless mice (Murakami et al., 2012). Therefore, it could conclude that both epidermis and dermis have abnormal amino acid metabolism disorders. Of note, phenylalanine and tyrosine are vital as precursors of melanin. Changes in these two amino acids in our study may be related to the excessive production and abnormal deposition of melanin stimulated by the inflammatory process of acne, resulting in pigment sequelae (Elbuluk et al., 2021). However, as quantitative analysis of these amino acids does not exist, a more detailed analysis of this pathway is warranted.

#### 4.1.2 Sphingolipid metabolism and sphingolipid signaling pathway

Our data demonstrated that Lipids and lipid-like molecules account for 25.51% of the identified differential metabolites between group M and group C, highlighting the correlation between aberrant lipid metabolism and acne. Among these lipid molecules, three sphingolipid metabolites, including sphingomyelin (SM) (d18:1/18:0), sphinganine, and sphingosine, were altered, indicating that the sphingolipid metabolism and sphingolipid signaling were significantly perturbed pathways in the rabbit ear acne model. Consistently, a previous study has identified the dysregulated sphingolipid metabolism in skin samples of the rat model of acne (Chen et al., 2021). Meanwhile, our previous study also found that the sphingolipid signaling pathway was the most altered in plasma of moderate-to-severe acne patients (Yu et al., 2022). Besides, SM can inhibit the differentiation of keratinocytes (Pillai et al., 1999). Sphingosine and sphinganine are potent promoters of keratinocyte differentiation (Paragh et al., 2008; Sigruener et al., 2013). Hence, decreased SM and increased sphingosine and sphinganine in our study can prompt the differentiation of keratinocytes, resulting in the thickening of the stratum corneum.

#### 4.1.3 ATP-binding cassette transporters

ABC transporters was another significant perturbed pathway in our study. ABC transporters are energy-dependent transmembrane transporters involved in regulating the transport of sugars, nucleosides, amino acids, fatty acids, lipids, and more (Behl et al., 2021a). In recent years a growing body of work has shed light on the connection between ABC transporters and lipid metabolism. ABC transporters can control the transport of lipid particles by opening and closing the transport cycle, which is involved in lipid homeostasis. This process is closely related to metabolic disorders (Behl et al., 2021b). The expression of ABC also inhibits sterol regulatory element binding protein, which inhibits adipogenesis (DeBose-Boyd and Ye, 2018). Dysregulation of ABC transporters results in excess free total cholesterol, which is detrimental to macrophages and adipocytes, thereby promoting programmed cell death (Wang and Westerterp, 2020). Besides, Palmer’s study showed that ABC transporters are highly expressed in sebaceous glands which role is to synthesise and secrete sebum (Palmer et al., 2021). Excessive sebum secretion has long been regarded as one of the critical factors in the pathogenesis of acne (Lu et al., 2019). Hence, the dysfunction of ABC transporters may be involved in the pathogenesis of acne by regulating the metabolism and transport of sebum.

### 4.2 Isotretinoin significantly reduces skin lipid metabolism in coal tar-induced acne in rabbits

Previous studies have shown that isotretinoin can alter human lipid metabolism by significantly reducing sebum production while raising blood lipids (Khabour et al., 2018). Gencebay’s study showed a 36% decrease in sebum levels after 6 months of oral isotretinoin systemic therapy in patients with acne vulgaris (Gencebay et al., 2021). Souza’s study also confirmed that low-dose oral isotretinoin significantly reduced sebum secretion in patients with seborrheic dermatitis (de Souza Leao Kamamoto et al., 2017). Consistently with their result, five lipids and lipid-like molecules, including LPC 18:1, PC 18:1, PC (16:0/16:0), alpha-Tocopherol, and Mesaconic acid, were normalized in group T.

Among them, PC 18:1, PC (16:0/16:0), and LPC 18:1 belong to the phospholipids. PC is a component of biological membranes, and its biosynthesis and degradation are considered essential for cell cycle processes, and its synthetic deficiency is a marker of apoptosis (Huang et al., 2020). Removing PC fatty acid chains at the sn-2 position via the action of cytosolic phospholipase A2 (cPLA2) results in the formation of LPC (Luczaj et al., 2020). LPC is biologically active lysophospholipids that have multiple stimulatory effects on various immune cells such as monocytes, macrophages, T lymphocytes, and neutrophils *in vitro* (Takatera et al., 2007). By binding to TLR2 and TLR4 receptors, LPC can activate NF-kB, p38MAPK, and JUN signaling pathways. Activation of these pathways can induce the production of pro-inflammatory factors such as IL-1β and IL-8 to regulate inflammatory and infectious diseases (Liu et al., 2020). Previous evidence demonstrated that IL-1β promotes the inflammatory response of P. acnes *in vitro* and *in vivo*, and IL-8 is also an essential pro-inflammatory cytokine in the pathogenesis of acne (Yang et al., 2021). Therefore, we hypothesize that the elevated PC and LPC identified in our study may be involved in the pathogenesis of acne by triggering TLR2 and TLR4-mediated signaling pathways to induce the production of inflammatory factors.

Vitamin E and mesaconic acid are both lipid-like molecules. Vitamin E is an essential component of human sebum, and sebaceous gland secretion is the relevant physiological pathway for delivering vitamin E to the upper layers of facial skin. This mechanism may protect skin surface lipids and the upper stratum corneum from harmful oxidation (Thiele et al., 1999). In our rabbit acne model, vitamin E levels increased significantly, which we believe can protect the skin from oxidation. Mesaconic acid is a methyl-branched fatty acid and can be produced by the breakdown of methylsuccinic acid and the squalene precursor farnesol (Coman et al., 2018; De Biase et al., 2019). Mesaconic acid is an isomer of itaconic acid, which has antibacterial, immunomodulatory, and cellular protective effects (Winterhoff et al., 2021). However, due to the limited research on mesaconic acid, the role of mesaconic acid in acne needs further study.

## 5 Conclusion

This work used UHPLC-qTOF-MS to explore the skin metabolomics and therapeutic effects of isotretinoin against acne. Through the analysis of the metabolic profiles, there were 98 biomarkers have been defined, which were closely relevant to the occurrence and development of acne. Metabolic pathways such as protein digestion and absorption, central carbon metabolism in cancer, ABC transporters, amino acid tRNA biosynthesis, amino acid biosynthesis, and sphingolipid signaling pathways were strongly associated with the development of acne. Besides, isotretinoin could normalize the dysregulation of lipid metabolites, such as LPC 18:1, PC 18:1, PC (16:0/16:0), alpha-Tocopherol, and Mesaconic acid. It serves as a new insight for further study of the pathogenesis of acne, and laying a foundation for finding new therapeutic targets of acne.

## Data Availability

The original contributions presented in the study are included in the article/Supplementary Materials, further inquiries can be directed to the corresponding author.
