# Supplementary figures and images for: Nontargeted metabolomics to characterize the effects of isotretinoin on skin metabolism in rabbit with acne
Source: Front Pharmacol. 2022 Aug 31;13:963472. doi: 10.3389/fphar.2022.963472 (PMC9470959; doi:10.3389/fphar.2022.963472)

# Supplementary Table S1 Differential metabolites identified in group C and group M

#
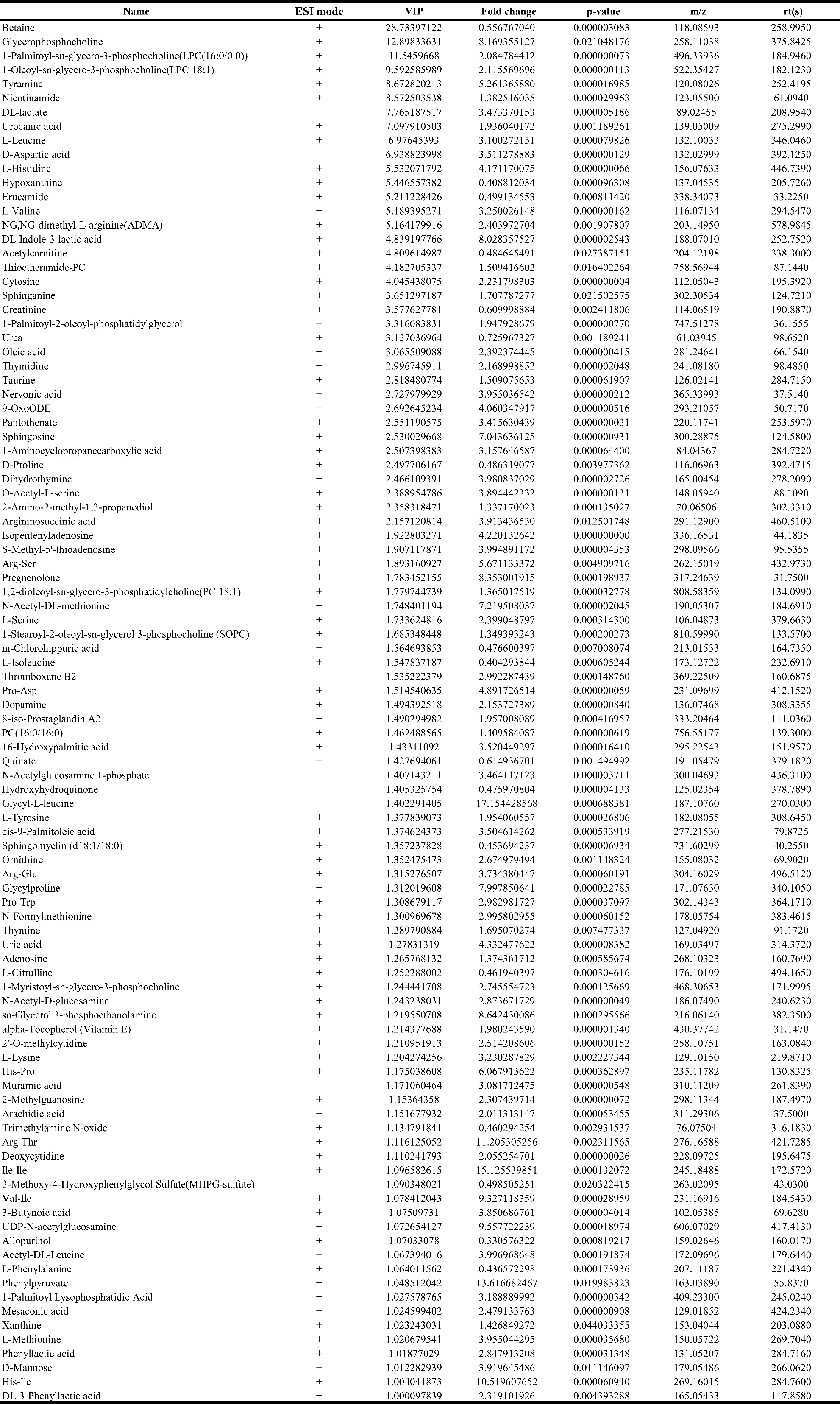

Supplement: Supplementary file 1 [file Table1.DOCX]

# Supplementary Table S2 ROC curves analysis of metabolites with VIP values in the top 10


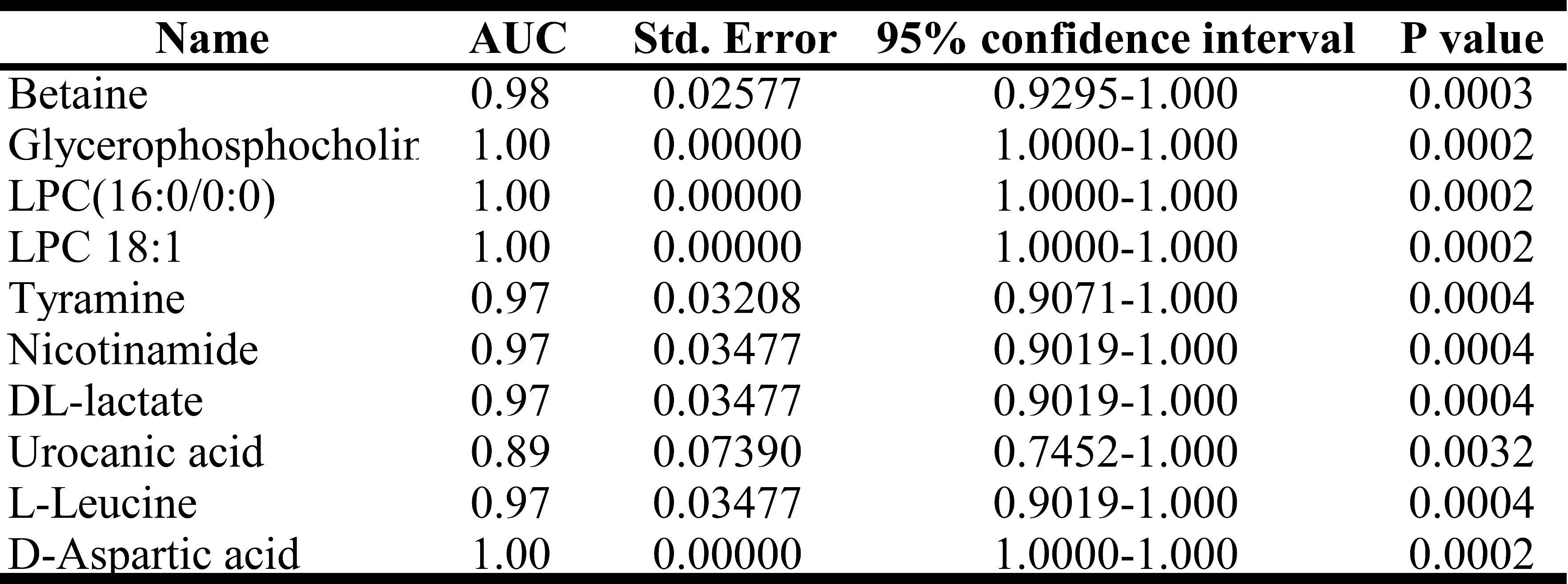

Supplement: Supplementary file 2 [file Table2.DOCX]

# Supplementary Table S3 Metabolic pathways that significantly changed in group C and group M
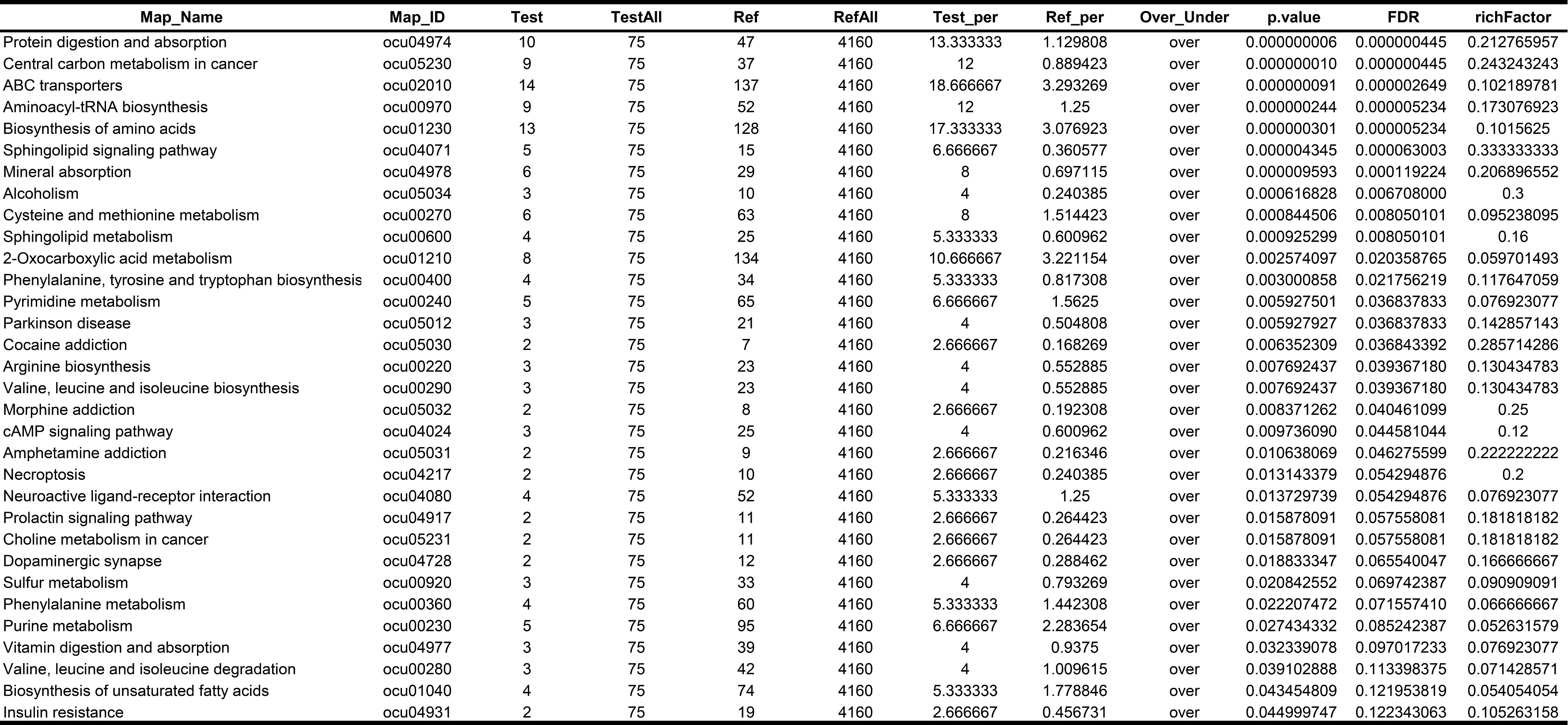

Supplement: Supplementary file 3 [file Table3.DOCX]
